# Exploring the Multifactorial Landscape: Risk Factors for Dementia in a Tertiary Care Setting in Thailand

**DOI:** 10.7759/cureus.60195

**Published:** 2024-05-13

**Authors:** Kasidid Lawongsa, Supatcha Kengpanich, Patsri Srisuwan

**Affiliations:** 1 Family Medicine, Phramongkutklao Hospital, Bangkok, THA; 2 Geriatrics, Phramongkutklao Hospital, Bangkok, THA

**Keywords:** tertiary care, risk factors, electronic medical record, survival analysis, dementia

## Abstract

Background: Dementia poses a significant public health challenge worldwide, necessitating a deeper understanding of its risk factors to inform preventive strategies.

Method: This retrospective longitudinal study leveraged clinical data from a tertiary care database to investigate the risk factors associated with an incident dementia diagnosis. The study cohort comprised individuals aged 50 years and older. Key variables including age, income, comorbidities such as depressive disorder, osteoporosis, stroke, and metabolic conditions like type 2 diabetes and hypertension were analyzed by using Cox regression analysis.

Result: The study cohort included 127,016 adults 50 years and older. The results revealed that advancing age, with individuals aged 70-79 years having a hazard ratio (HR) of 3.9 (95% confidence interval (CI), 2.6-5.8), and those aged 80 years and above having an HR of 11.6 (95% CI, 7.7-17.3), lower income status (patients with no income or occupation had a notably higher risk of dementia diagnosis, with an HR of 2.0 (95% CI, 1.4-2.8)), depressive disorder (HR of 3.3 (95% CI, 3.3-3.7)), osteoporosis (HR of 1.2 (95% CI, 1.1-1.4)), and stroke (HR of 2.5 (95% CI, 2.3-2.7)) were significantly associated with an increased risk of incident dementia. However, no significant associations were observed for type 2 diabetes, hypertension, obesity, or underweight status managed in tertiary care.

Conclusion: The findings underscore the importance of considering a wide range of factors in understanding dementia risk and highlight the potential utility of routinely collected clinical data for comprehensive risk assessment. Further investigation into additional variables and multi-center studies may provide deeper insights into the complex interplay of risk factors contributing to dementia onset.

## Introduction

Dementia, an umbrella term for progressive neurodegenerative conditions, has become a global health priority due to its increasing prevalence [1]. Marked by cognitive decline disrupting daily life, dementia poses significant challenges globally [2]. To grasp dementia fully, we must look beyond its clinical surface.

Thailand, like many nations experiencing a demographic shift toward an aging population, faces a growing concern with dementia. Studies suggest a significant portion of the elderly population may be affected, with estimates ranging from 1.8% to 10.2% among those over 55 years old [3]. Notably, the prevalence increases dramatically with age. A national survey found a mere 1% prevalence in the 60-64 age group, but this figure soars to a concerning 31.3% for those aged 90 and above [4].

Dementia, an escalating neurodegenerative condition marked by a cognitive decline hindering daily functioning, poses a significant global health dilemma [1]. Although aging remains the primary risk factor, dementia’s onset is influenced by a mix of modifiable and non-modifiable factors [5]. Current evidence from epidemiological studies and clinical research indicates that numerous factors beyond aging contribute to the risk of developing dementia. These encompass cardiovascular health, diabetes, hypertension, physical activity, dietary habits, cognitive engagement, socioeconomic status, and environmental influences. While some factors like age and genetic predisposition are non-modifiable, others such as lifestyle choices and specific health behaviors can be modified through interventions. It is crucial to comprehend how these factors interact and influence dementia risk to formulate effective preventive measures and interventions customized to the needs of individuals and communities. Understanding these risks is crucial for bolstering brain health, possibly delaying dementia onset, and shaping preventive measures.

This research investigates the presence of pre-diagnostic risk factors for dementia utilizing the Phramongkutklao Hospital database (PHD). It aims to quantify the potential impact of modifiable risk factors, comorbidities, and demographic characteristics on dementia incidence among individuals aged 50 years and above in the tertiary outpatient department. By leveraging routinely collected clinical data, this study addresses challenges encountered by clinical trials and prospective cohort designs in achieving representative study samples [6]. It seeks to mitigate selection bias, ensuring inclusion of patients with varying health conditions or dementia risk levels, and information bias, accounting for difficulties in symptom recall and response among patients with cognitive decline. This inquiry will explore its diverse forms, underlying neuropathology, and the intricate web of risk factors contributing to its emergence. We'll scrutinize Alzheimer's disease, the most prevalent type, alongside other common forms, unraveling the biological processes driving neuronal degeneration [7].

## Materials and methods

Study design

This study adopted a retrospective cohort design, utilizing electronic medical record (EMR) data from the PHD. The study population comprised individuals aged 50 years and above, with recorded demographic information (gender, age, income). The inclusion criteria were individuals aged 50 years and older, absence of a pre-existing dementia diagnosis at baseline, availability of relevant health records or medical history, and residency within Thailand. The exclusion criteria for the study were individuals aged under 50 years; those with a history of smoking or alcohol addiction; and patients with incomplete medical records, including missing data.

To ensure the accuracy of incident cases, 2009 was designated as a wash-out year; individuals diagnosed with dementia before December 31, 2008, were classified as prevalent cases and excluded from the analysis. The study's index date was set as January 1, 2009, and the endpoint was determined as the earliest of either the dementia diagnosis date, two years after the last recorded visit, or December 31, 2023 or until they experienced loss to follow-up, mortality, or volunteer withdrawal from the healthcare system.

Data source

The PHD serves as a platform for EMR-based tertiary care research. Biannually, deidentified patient-level clinical data, comprising diagnoses, prescribed medications, demographics, medical examinations, laboratory test results, referrals, and risk factors, is extracted from primary care EMRs supported by diverse vendors and standardized for analysis.

As of 2023, the PHD contained records of 127,016 patients aged 50 years and above. These figures may suggest that older adults, with their heightened health concerns, tend to seek care from family physicians more frequently. In the PHD, the sex ratio among individuals aged 50 years and above is approximately 0.89 males to females.

Case definition

Dementia

The PHD employs a comprehensive algorithm selects random samples from each type of dementia with similar demographic characteristics and roughly equal numbers as its case definition for dementia, aiming to encompass all its various types. It mandates evidence from health condition records (or problem lists), encounter diagnoses, or billing tables indicating an ICD-10 code of F00-F03, G30, or a prescription for a cholinesterase inhibitor (Rivastigmine, Galantamine, or Donepezil) or an N-methyl-D-aspartate (NMDA) receptor antagonist (Memantine).

The date of dementia diagnosis is established as the clinical encounter recorded in the EMR when a patient initially meets the validated PHD case criteria for the condition.

Covariates

The study considered the following variables: age (≥50 years), sex (male or female), weight, height, and income. Baseline comorbidities were identified using ICD-10-CM codes and Anatomical Therapeutic Chemical (ATC) codes for medications. These comorbidities included type 2 diabetes (ICD-10-CM code: E11), hypertension (ICD-10 codes: I10-I15), stroke (ICD-10-CM codes: I60-I66), depressive disorder (ICD-10-CM codes: F32-F33), osteoporosis and osteoporotic fractures (ICD-10-CM codes: M80-M82), estrogen supplementation (ATC codes: T385, Y425, EST103N, PRO108E, ANG103N), and bisphosphonates (ATC codes: ALE104E, FOS103N, BON103N, ACT107N, RIS108N, ACL201N, ZOL203N, ZOL202N, PRO213N, XGE200N, CEL104N, MIA201N, FOR202N, EVE201N), which are primary treatments for osteoporosis.

Statistical analysis

All statistical analyses were conducted using IBM SPSS Statistics software (version 26.0; IBM Corp., Armonk, NY, USA). The relationships between variables and osteoporosis were assessed using the chi-square test. Comparisons of demographics and dementia diagnosis between the two groups were made. Clinical variables were examined using independent t-tests, and chi-square tests.

Survival analysis employing the Cox proportional hazard model was conducted to estimate hazard ratios (HRs). The denominators for person-years at risk were calculated as the total number of years from the index date to the last eligible date for each participant. The likelihood ratio test yielded a non-significant result (χ² = 15.42, df = 10, p = 0.12), suggesting that the model adequately fits the data.

Ethics

The PHD database obtained ethics approval from the Institutional Review Board of the Royal Thai Army Medical Department, which included waivers of individual patient consent for the utilization of their deidentified data for surveillance and research purposes. This study obtained specific approval from the Institutional Review Board of the Royal Thai Army Medical Department (IRBTA0269/2567).

## Results

In the study population, there were 22,859 (18.0%) patients aged 50-59 years, 39,051 (30.7%) aged 60-69 years, 34,777 (27.4%) aged 70-79 years, and 30,329 (23.9%) aged 80 and above. The older age group exhibited a higher proportion of females (52.7% versus 47.3%, P < 0.001) and fewer individuals with recorded income (78.6% versus 21.4%, P < 0.001). The prevalence of osteoporosis was 8.4%, type 2 diabetes was 10.2%, stroke was 5.7%, and depressive disorder was 3.1%. Hypertension, however, had a higher prevalence in the older age group. Table 1 provides an overview of the baseline characteristics of the two age groups and the entire study cohort.

**Table 1:** Demographic and health characteristics of patients at baseline. The data has been represented as N, %, $Mean\pm SD$. * P<0.001, ** P<0.05.

Characteristic		Total		Dementia				P-value
				No		Yes		
		n	%	n	%	n	%	
All		127,016	-	121,612	95.7	5,404	4.3	-
Sex	Male	60,138	47.3	58,583	97.4	1,555	2.6	<0.001*
	Female	66,878	52.7	53,516	80.0	13,362	20.0	
Age	50-59	22,859	18.0	22,734	99.5	125	0.5	<0.001*
	60-69	39,051	30.7	38,531	98.7	520	1.3	
	70-79	34,777	27.4	33,417	96.1	1,360	3.9	
	80+	30,329	23.9	26,930	88.8	3,399	11.2	
Income	No	99,879	78.6	94,632	94.7	5,247	5.3	<0.001*
	Yes	27,136	21.4	26,980	99.4	156	0.6	
Osteoporosis	No	116,343	91.6	111,516	95.9	4827	4.1	<0.001*
	Yes	10,673	8.4	10,096	94.6	577	5.4	
Type 2 diabetes	No	114,112	89.8	109,205	95.7	4907	4.3	0.003*
	Yes	12,904	10.2	12,474	96.7	430	3.3	
Hypertension	No	9,957	7.8	8,642	86.8	1315	13.2	<0.001*
	Yes	117,059	92.2	112,970	96.5	4089	3.5	
Stroke	No	119,759	94.3	115,197	96.2	4562	3.8	<0.001*
	Yes	7,257	5.7	6,415	88.4	842	11.6	
Depressive disorder	No	123,082	96.9	118,133	96.0	4949	4.0	<0.001*
	Yes	3,934	3.1	3,479	88.4	455	11.6	
Estrogen suplementation	No	126,447	99.6	121,060	95.7	5387	4.3	<0.001*
	Yes	569	0.4	552	97.0	17	3.0	
Calcium supplementation	No	66,408	52.3	64,062	96.5	2346	3.5	<0.001*
	Yes	60,608	47.7	57,550	95.0	3058	5.0	
BMI (mean±sd)		24.68±4.26		24.75±4.25		23.05±4.09		<0.001*

**Figure 1:**
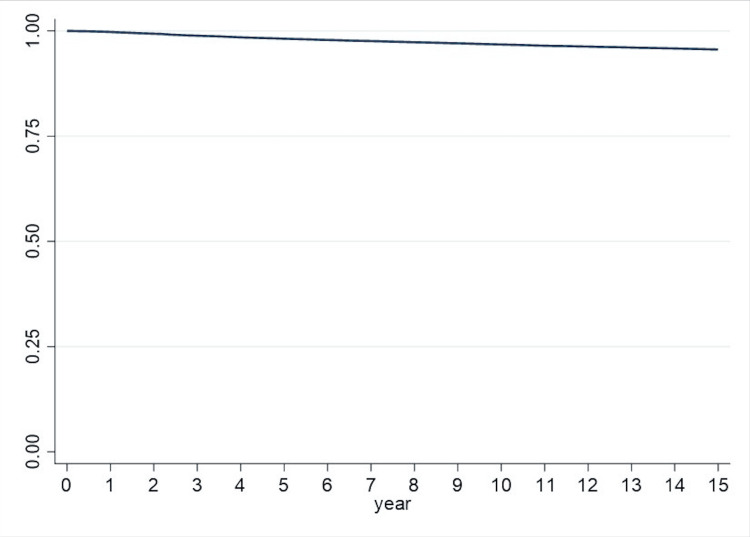
Kaplan-Meier curves of dementia-free survival within 15 years from the index date.

Among patients aged 50 years and older, there were 5,404 cases of dementia. After adjusting for comorbidities and demographic characteristics, the risk of dementia onset escalated with age, with individuals aged 70-79 years exhibiting an HR of 3.9 (95% confidence interval (CI), 2.6-5.8), and those aged 80 years and above having an HR of 11.6 (95% CI, 7.7-17.3). Patients with no income or occupation had a notably higher risk of dementia diagnosis, with an HR of 2.0 (95% CI, 1.4-2.8). Depressive disorder, osteoporosis, and stroke significantly heightened the risk of incident dementia (P-value < 0.001). Patients diagnosed with depression faced a higher risk, with an HR of 3.3 (95% CI, 3.3-3.7), compared to those without depression. Similarly, patients with osteoporosis were at a heightened risk compared to those without, with an HR of 1.2 (95% CI, 1.1-1.4), and patients with stroke exhibited a higher risk compared to those without, with an HR of 2.5 (95% CI, 2.3-2.7). However, sex, type 2 diabetes, hypertension, obesity, and underweight did not show statistically significant associations with dementia onset. Table 2 outlines the adjusted HRs of dementia onset for both age groups. Kaplan-Meier curves (Figure 1) were utilized to analyze dementia-free survival within 15 years from the index date. Statistical significance was determined with a threshold of p < 0.05.

**Table 2:** Hazard ratios of the onset of dementia. "1" serves as the reference group, * P<0.001, 95%CI - 95% confidence interval.

Characteristic		Crude Hazard Ratio	95%CI	P-value	Adjusted Hazard Ratio	95%CI	P-value
								Age	50-59	1	-	-	1		
									60-69	2.49	2.05-3.03	<0.001*	1.34	0.91-1.99	0.143
70-79	7.58	6.31-9.10	<0.001*	3.87	2.57-5.80	<0.001*		
80+	22.95	19.20-27.44	<0.001*	11.57	7.72-17.34	<0.001*		
Income	No	9.71	8.28-11.38	<0.001*	1.95	1.36-2.81	<0.001*
	Yes	1	-	-	1	-	-
Depressive disorder	No	1	-	-	1	-	-
	Yes	2.99	2.71-3.29	<0.001*	3.34	3.04-3.68	<0.001*
Osteoporosis	No	1	-	-	1	-	-
	Yes	1.79	1.65-1.96	<0.001*	1.23	1.10-1.37	<0.001*
Stroke	No	1	-	-	1	-	-
	Yes	3.14	2.92-3.38	<0.001*	2.48	2.31-2.67	<0.001*

## Discussion

This study investigated the incidence of dementia in relation to various demographic and health factors. Our findings align with previous research indicating a significant association between advancing age and an elevated risk of dementia onset. This consistent pattern underscores the critical role of age as a fundamental factor influencing dementia risk [8]. Additionally, our study identified several other factors associated with an increased risk of dementia, including a history of no income, depressive disorder, osteoporosis, and stroke. These findings are consistent with prior studies that have demonstrated similar associations between these factors and dementia risk. For example, research has shown that late-life depression may predispose individuals to a higher risk of developing dementia, possibly through neurobiological mechanisms involving inflammation and neurodegeneration [9,10]. Similarly, osteoporosis has been linked to an elevated risk of dementia, possibly due to shared underlying pathophysiological processes, such as chronic inflammation and vascular dysfunction [11,12]. Additionally, the association between stroke and dementia is well-established, with cerebrovascular disease contributing to cognitive decline and the development of dementia, particularly vascular dementia (VaD) [13]. However, our study did not find significant associations between dementia risk and other factors such as type 2 diabetes mellitus (T2DM), hypertension, obesity, or being underweight. These findings contrast with some previous studies, highlighting the complexity of the relationship between these factors and dementia risk. Potential explanations for the lack of significant associations in our study include the presence of other confounding variables, the heterogeneity of dementia subtypes, and differences in study populations and methodologies. Further research is needed to elucidate the underlying mechanisms and explore potential interactions between these factors to better understand their role in dementia risk.

Type 2 diabetes

Rusanen et al. [14] conducted a study investigating the association between T2DM and the risk of Alzheimer’s disease (AD) and VaD, while Cheng et al. [15] conducted a meta-analysis on the same topic. Surprisingly, both studies found no significant association between T2DM and either AD or VaD after adjusting for various confounding factors. Several factors may contribute to the lack of a consistent association, including the presence of other vascular risk factors often accompanying T2DM, such as hypertension and dyslipidemia, which can independently contribute to dementia development. Moreover, the heterogeneous nature of dementia, encompassing various subtypes like AD and VaD, complicates establishing a direct link with T2DM. Additionally, differences in study populations, methodologies, and diagnostic criteria across studies may also contribute to conflicting findings. The underlying mechanisms linking T2DM to dementia remain unclear, although hyperglycemia, insulin resistance, and chronic inflammation have been proposed as potential contributors. It is plausible that T2DM may influence dementia risk through indirect pathways, such as promoting cerebrovascular disease or exacerbating neurodegenerative processes.

Hypertension

In a study by Gottesman et al. [16], the relationship between midlife hypertension and late-life cognitive decline was investigated, revealing surprising results that midlife hypertension was not significantly associated with an increased risk of dementia or cognitive impairment in later life. Similarly, a systematic review and meta-analysis by Deckers et al. [17] also failed to find clear evidence of a causal relationship between hypertension and dementia. Several factors may contribute to the lack of a consistent association between hypertension and dementia. Firstly, hypertension often coexists with other vascular risk factors like diabetes and dyslipidemia, which can independently contribute to cognitive decline. Additionally, the precise mechanisms underlying the association between hypertension and dementia remain unclear. While hypertension may lead to cerebrovascular disease and VaD through the disruption of cerebral blood flow and the promotion of atherosclerosis, its role in the development of AD, the most common form of dementia, is less understood.

Underweight and overweight

Research investigating the connection between body weight and the risk of dementia has produced conflicting results, with some studies indicating possible associations while others failed to establish a significant link. For example, Albanese et al. [18] explored the correlation between body mass index (BMI) and dementia risk, concluding that neither underweight nor overweight status significantly impacted dementia incidence. Similarly, Pedditizi et al. [19] conducted a systematic review and meta-analysis, revealing inconclusive evidence regarding the relationship between BMI and dementia risk, with varying findings across studies. The inconsistency in results may stem from the multifaceted nature of dementia etiology, involving genetic, environmental, and lifestyle factors, complicating efforts to pinpoint the specific influence of body weight. Furthermore, confounding variables like comorbidities, socioeconomic status, and physical activity levels further obscure the interpretation of study outcomes. Additionally, while obesity has been theorized to contribute to dementia risk through associations with cardiovascular risk factors and metabolic dysfunction, the impact of being underweight on dementia risk remains unclear. Further investigations are warranted to better comprehend the intricate interplay between body weight, metabolic well-being, and the risk of dementia.

Limitation

In conducting a retrospective longitudinal follow-up study using available and accessible information within the PHD-processed EMR data, our research drew from a non-representative sample of the Thailand population as it focused solely on a single center population. However, the secondary use of clinical data is constrained by the availability and quality of the information, which was initially collected by healthcare providers for non-research purposes. This limitation could introduce information bias, particularly if cases of dementia were misclassified or misdiagnosed. However other information biases like recall bias or interviewer bias are expected to be minimal as data were routinely entered by clinicians during healthcare provision. Moreover, our study is susceptible to the risk of unmeasured confounding, as the current database limitations hinder the accurate evaluation of competing risks from potential factors in dementia incidence, such as smoking, inherited genetic conditions, physical inactivity, and mental illnesses other than depression, as well as mortality. Additionally, other medications like prescription opioids and benzodiazepines, which could serve as potential confounders, were not considered in our analysis. Further investigation into the relationship between the use of drugs to treat chronic pain or anxiety and dementia onset, established in a multi-center setting, would be beneficial. Another limitation is the inability to distinguish dementia subtypes in this study due to the current lack of subtype case definitions. The ability to differentiate between VaD, AD, and other forms of dementia could contribute to a more nuanced understanding of risk factors.

## Conclusions

This study revealed that age 70 years and more, income, depressive disorder, osteoporosis, and stroke were linked to a heightened risk of incident dementia diagnosis. However, T2DM, hypertension, obesity, or underweight, managed in tertiary care, did not exhibit such an association. Further exploration into the relationship between laboratory test results, medications, or other treatment activities recorded across multiple centers may prove beneficial in elucidating the apparent association between other chronic diseases and the incidence of dementia.
